# Impact of COVID-19-like symptoms on occurrence of anxiety/depression during lockdown among the French general population

**DOI:** 10.1371/journal.pone.0255158

**Published:** 2021-07-26

**Authors:** Murielle Mary-Krause, Joel José Herranz Bustamante, Mégane Héron, Astrid Juhl Andersen, Tarik El Aarbaoui, Maria Melchior

**Affiliations:** Sorbonne Université, INSERM, Institut Pierre Louis d’Épidémiologie et de Santé Publique (IPLESP), Equipe de Recherche en Epidémiologie Sociale (ERES), Paris, France; Fukushima Medical University School of Medicine, JAPAN

## Abstract

**Background:**

The outbreak of the COVID-19 epidemic lead to high levels of morbidity and mortality around the globe. Consequences of this outbreak and possible associated infection are an increase in mental health disorders and an increased likelihood of internalizing problems, particularly depression. However, to date few studies have tested this hypothesis while taking into account individuals’ preexisting mental health difficulties.

**Methods:**

We used longitudinal data collected among 729 persons in the context of the French TEMPO cohort between March and June 2020 (7 waves of data collection). COVID-19-like symptoms as well as anxiety/depression (assessed by the Adult Self Report), were reported at each wave of data collection. To study the relationship between COVID-19-like symptoms and anxiety/depression, we used generalized estimation equation (GEE) models controlled for socio-demographic and health-related characteristics, including anxiety/depression prior to 2020.

**Results:**

Overall, 27.2% of study participants reported anxiety/depression during lockdown. 17.1% of participants reported COVID-19-like symptoms during the course of follow-up, 7.3% after the beginning of lockdown, with an average number of 2.7 symptoms, and 3.6% reported respiratory distress. In multivariate analyses, nearly all the considered indicators of COVID-19-like symptoms were associated with higher odds of symptoms of anxiety/depression (symptoms Yes/No: OR = 1.66, 95% CI = 1.08–2.55; symptoms after the beginning of lockdown: OR = 1.91, 95% CI = 1.03–3.52; number of symptoms: OR for each additional symptom = 1.19, 95% CI = 1.02–1.39. This relationship exists after taking into account prior symptoms of anxiety/depression, which are associated with a 5-fold increased likelihood of psychological distress. And this impact is stronger among men than women.

**Conclusions:**

Our study shows higher risk of anxiety/depression among persons who experienced COVID-19-like symptoms, even after accounting for prior mental health difficulties. COVID-19 infection could have both a direct and indirect impact on the occurrence of psychological difficulties, and this association should be studied in greater detail.

## Introduction

The COVID-19 outbreak, caused by the SARS-CoV-2 virus and the associated public health control measures have been shown to be associated with increased mental health disorders in the general population [1, 2]. Prevalence of mental health problems due to COVID-19 infection in the general population varies depending on the study population and the setting of the COVID-19 outbreak [3]. At the beginning of the outbreak, anxiety and depression were respectively observed among 28.8% and 16.5% in the Chinese population [4]. Systematic reviews indicate that in general population samples, the prevalence of anxiety symptoms ranges from 6.3% to 50.9% and that of depressive symptoms from 14.6% to 48.3% [5, 6]. This high heterogeneity could be due to differences in studied countries, the epidemic conditions of COVID-19 in under-developed and developing countries imposing greater psychological effects on the population and because uncertainty about health status and care increasing the vulnerability of such communities [5]. The time when prevalences were estimated may also explain the differences as the highest levels of anxiety and depression were shown to occur in the early stages of lockdown and declined fairly rapidly after [7]. This could also be due to the sampling method introducing possible bias, due to differences in sex, age or levels of education distribution in the studied population. Among young adults (18–24 years), a study conducted in the US reported a prevalence of 49.1% for anxiety disorders and 52.3% for depressive disorders [8], while another investigation conducted among adolescent athletes reported a prevalence of moderate and severe anxiety symptoms of 20.1% and 16.6% respectively [9]. In France, the prevalence of anxiety and depressive symptoms during lockdowns (March 17-May 11, 2020 and October 30- December 15, 2020, respectively) were around 20% in the general population [10].

Risk factors of mental health problems during the COVID-19 epidemic include socio-demographic characteristics such as female sex, younger age and lower educational and socioeconomic status [4, 6], but also pre-existing mental illness [11–13], self-rated health status and social isolation [6]. Moreover factors linked to the COVID-19 infection such as related symptoms, contact with persons affected with COVID-19, COVID-19-related fear, as well as the level of information regarding COVID-19, have also been found to contribute to the deterioration of mental health [4, 6, 11–15]. However, most of these studies were cross-sectional [6], and some associations were found only in univariate models [4, 12].

Moreover, even though many studies have reported increased anxiety and depression as a consequence of the COVID-19 epidemic, individuals’ pre-pandemic history of mental health problems was mostly not taken into account, with the exception of few studies for which this information was collected retrospectively and may therefore have been tainted by recall bias [11–13]. Some studies tried to compare prevalences before and after the COVID-19 epidemic. They showed that the prevalence of symptoms of anxiety and depression in 2020 is higher than in the same period in 2019 [16], 2018 [17], or before the pandemic [18]. So, direct evidence of COVID-19 infection as a risk factor associated with an increase in mental health risk is limited. Moreover, mental health surveys conducted during the COVID-19 pandemic most frequently relied upon convenience samples, which are prone to substantial bias [19]. Nevertheless, a recent study in 3 Dutch cohorts comparing people with and without mental health disorders [20], showed that, compared to periods prior to COVID-19, symptoms of depression and worry increased during the pandemic, with no significant changes in symptoms of anxiety, and no overall increase in symptom severity in individuals with the largest mental health disorder burden. Thus, while it may be that preexisting mental health difficulties increase risk of poor mental health during the COVID-19 pandemic, this hypothesis has not been fully supported by existing data, and requires to be examined by taking into account risk factors specific to this period, including COVID-19 infection itself and consequences of this infection.

To limit COVID-19 transmission, France established a first lockdown from March 17^th^ to May 12^th^ 2020. The objective of this study is to examine the associations between different measures of the COVID-19 symptomatology and anxiety/depression during the first COVID-19 lockdown in a cohort of middle-aged adults in France, taking into account other risk factors including comorbidity, prior mental health problems and socio-economic characteristics. We hypothesize that the relationship between COVID-19-like symptoms and anxiety/depression exists even when prior mental health problems are taken into account.

## Methods

### Study population

The TEMPO COVID-19 project derives from the TEMPO (Trajectoires ÉpidéMiologiques en POpulation) cohort, a study set up in 2009 to evaluate individual, familial and social determinants of mental health difficulties, addictive behaviors and their trajectories over time. TEMPO cohort participants took part in a study of children’s psychological problems and access to mental healthcare in 1991 (age 4–16 years, *n* = 2,582) and were followed-up via self-completed questionnaires in 1999, 2009, 2011, 2014 and 2018 [21]. Since 1991, a total of 3,401 persons participated in at least one wave of data collection, responding to the study questionnaire either online or via postal questionnaire.

The TEMPO COVID-19 project comprises 7 waves of data collection starting March 24^th^ 2020, one week after the beginning of the first COVID-19-related lockdown in France, until one week after the end of the lockdown, May 12^th^ 2020. Data were collected via self-completed electronic surveys sent out to the 1224 TEMPO cohort participants with a valid email address. Data were collected weekly for the first 5 surveys and fortnightly for the 6^th^ and 7^th^ surveys (**S1 Table**). Overall, 729 individuals completed at least one study questionnaire between March 24 and June 06, 2020.

The TEMPO cohort received approval from the French national committee for data protection (Commission National Informatique et Liberté, CNIL, n° 908163). In accordance with French regulations when the study was implemented, participants were informed that they had the right to refuse participation or withdraw from the study.

### Measures

#### Outcome variable: Symptoms of anxiety/depression

Participants’ symptoms of anxiety/depression were assessed using the specific subscale of the Adult Self Report (ASR) Achenbach System [22, 23]. Eight items were included in wave 1 [23] and 13 items in waves 2 to 7 [24] of the TEMPO COVID-19 project (**S2 Table**). Each item is scored 0 to 2 and the level of symptoms is calculated by summing all relevant items. To be rendered comparable between waves, ASR scores were standardized from 0 to 100. Following ASR guidelines, we dichotomized the score using the 85^th^ percentile, people with higher scores being considered anxious/depressed [25].

#### Exposure variables: COVID-19-like symptoms

In each wave of the TEMPO COVID-19 questionnaire, participants were asked whether they had experienced COVID-19-like symptoms and if yes, which ones (from a list including: fever, cough, muscle soreness, respiratory problems, loss of taste, loss of smell, fatigue) and the date at which these symptoms started. In the first questionnaire, we asked about the presence of COVID-19-like symptoms at any point in time. From the 2^nd^ questionnaire onwards we only asked about the presence of COVID-19-like symptoms in the preceding 7 days. Based on this information, we derived four measures: 1) the presence of COVID-19-like symptoms (yes vs. no), 2) the timing of COVID-19-like symptoms (none, before the beginning of lockdown (< March 17, 2020), after the beginning of lockdown (≥March 17, 2020), 3) the number of COVID-19-like symptoms, 4) the type of COVID-19-like symptoms (none, mild, respiratory distress).

#### Covariates

Covariates included participants’ demographic, socioeconomic and health characteristics. Level of education was divided into 3 categories: “≤High School degree”, “2 to 4 years university degree”, and “≥5 years university degree”. Household configuration during lockdown was divided into 3 categories: “Living with a partner + children”, “Living with a partner (without children)” and “Other”. Household income level/month during lockdown was dichotomized in two categories: “>2500 €” vs. “≤2500 €”. Employment stability during lockdown was defined as follows: “Permanent contract or civil servant” or “Self-employed” were considered as stable, any other occupational status was considered as unstable. Working status during lockdown was considered using three categories: “Working from home / Changed working patterns”, “Working as usual” and “Unemployed”.

History of anxiety/depression prior to 2020 was assessed using TEMPO data collected in 2009, 2011 or 2018. For each individual, the most recent information available was taken into account. Anxiety/depression was assessed by the ASR in 2009 and 2018 and by the Mini-International Neuropsychiatric Interview (MINI) [26] in 2011. Scores from different scales were standardized from 0 to 100 and dichotomized according to the 85^th^ percentile of the most recent information available, corresponding to a mean score of 34. Persons who participate more actively in the survey are more likely to be in the latter wave resulting in a possible bias. So, a sensitivity analysis was conducted using the first available (oldest) data to establish whether participants had a history of anxiety/depression prior to 2020, instead of the most recent information.

### Statistical analysis

To study the relationship between participants’ COVID-19-like symptoms and anxiety/depression, we proceeded as follows. First, we described sample characteristics according to whether participants reported COVID-19-like symptoms or not. No collinearity between all variables was observed. Second, we tested bivariate and multivariate associations between COVID-19-like symptoms and anxiety/depression using generalized estimation equation (GEE) models with a logit link, binomial distribution and unstructured correlation matrix. Four different GEE models were implemented, one for each of the four measures of COVID-19-like symptoms, retaining all covariates found to have a p-value<0.2 in bivariate analyses. Third, interactions between COVID-19-like symptoms and a) sex, b) income level, c) diabetes and/or overweight/obesity, d) anxiety/depression prior to 2020 and all variables included in the final models were tested. All analyses were carried out using SAS^®^ 9.4.

## Results

Among the 729 participants to the TEMPO COVID-19 project, 27.2% (n = 195) reported symptoms of anxiety/depression during lockdown. COVID-19-like symptoms were reported by 17.1% of participants (n = 125) during the course of follow-up, 7.3% (n = 53) after the beginning of lockdown, with an average number of 2.7 symptoms (standard deviation SD = 1.5), and 3.6% (n = 26) had respiratory distress. **Table 1** presents participants’ characteristics according to whether they reported COVID-19-like symptoms or not and **Table 2** provides the results of all four bivariate GEE models for each measure of COVID-19-like symptoms. COVID-19-like symptoms were associated with anxiety/depression regardless of the indicator considered: any COVID-19-like symptoms, COVID-19-like symptoms after lockdown, number of COVID-19-like symptoms, and mild symptoms as well as respiratory distress. After adjustment (**Table 3**), any COVID-19-like symptoms remained associated with anxiety/depression during lockdown (66% increased odds), as were COVID-19-like symptoms after the beginning of lockdown (91% increased odds), the number of COVID-19-like symptoms (19% increased odds for each additional symptom). All types of COVID-19-like symptoms were not found associated with anxiety/depression but the significance is close: mild symptoms: OR = 1.71, 95% CI = 0.96–3.05, respiratory distress: OR = 1.96, 95% CI = 0.93–4.15. In the sensitivity analysis using the first rather than the latest available information on participants’ history of anxiety/depression, results are very similar and the conclusions the same **(S3 Table)**.

**Table 1:** Characteristics of TEMPO cohort participants during the first COVID-19-related lockdown (March-June 2020, France, % or mean*±* Standard Deviation (SD)).

		Total (n = 729) n (%)	Absence of COVID-19-like symptoms (n = 604) n (%)	Presence of COVID-19-like symptoms (n = 125) n (%)	p-value
***Socio-demographic characteristics***					
Sex (n = 729)					
	*Male*	257 (35.3%)	220 (36.4%)	37 (29.6%)	0.1461
	*Female*	472 (64.7%)	384 (63.6%)	88 (70.4%)	
Age (n = 729)					
	*Mean ± SD*	39.3 ± 3.6	39.3 ± 3.6	39.3 ± 3.9	0.8971
	*Median (Q1—Q3)*	40 (37–42)	40 (37–42)	40 (37–42)	
Highest level of education (n = 728)					
	*≤High School*	76 (10.4%)	65 (10.8%)	11 (8.8%)	0.5495
	*2 to 4 years university degree*	310 (42.6%)	260 (43.1%)	50 (40.0%)	
	*≥5 years university degree*	342 (47.0%)	278 (46.1%)	64 (51.2%)	
Household Configuration (n = 714)					
	*Living with a partner + children*	461 (64.6%)	390 (65.9%)	71 (58.2%)	0.2439
	*Living with a partner*	78 (10.9%)	61 (10.3%)	17 (13.9%)	
	*Other*	175 (24.5%)	141 (23.8%)	34 (27.9%)	
Household Income level/month (n = 695)					
	*> 2500 €*	566 (81.4%)	468 (81.4%)	98 (81.7%)	0.9437
	≤ *2500 €*	129 (18.6%)	107 (18.6%)	22 (18.3%)	
Employment stability (n = 706)					
	*Yes*	644 (91.2%)	531 (91.1%)	113 (91.9%)	0.7787
	*No*	62 (8.8%)	52 (8.9%)	10 (8.1%)	
Working status during lockdown (n = 717)					
	*Working from home/Changed working patterns*	447 (62.4%)	375 (63.0%)	72 (59.0%)	0.6004
	*Working as usual*	112 (15.6%)	93 (15.6%)	19 (15.6%)	
	*Unemployed*	158 (22.0%)	127 (21.4%)	31 (25.4%)	
***Health-related characteristics***					
Diabetes and/or Overweight-Obesity (n = 729)					
	*No*	640 (87.8%)	529 (87.6%)	111 (88.8%)	0.7052
	*Yes*	89 (12.2%)	75 (12.4%)	14 (11.2%)	
Contacts with COVID-19 infected persons (n = 729)					
	*No*	578 (79.3%)	495 (82.0%)	83 (66.4%)	**< .0001**
	*Yes*	151 (20.7%)	109 (18.0%)	42 (33.6%)	
Anxiety/depression prior to 2020 (n = 719)					
	*No*	533 (74.1%)	449 (75.1%)	84 (69.4%)	0.1946
	*Yes*	186 (25.9%)	149 (24.9%)	37 (30.6%)	
Anxiety-depression during lockdown (n = 719)					
	*No*	524 (78.8%)	448 (75.3%)	76 (61.3%)	**0.0014**
	*Yes*	195 (27.2%)	147 (24.7%)	48 (38.7%)	

**Table 2:** COVID-19-like symptoms and anxiety/depression in the TEMPO cohort during the first COVID-19-related lockdown (March-June 2020, France, bivariate GEE models, Odds-Ratio (OR), 95% Confidence Interval (CI)).

Measures of COVID-19-like symptoms		Model 1 (n = 719)	Model 2 (n = 717)	Model 3 (n = 681)	Model 4 (n = 681)
		OR [95% CI]	OR [95% CI]	OR [95% CI]	OR [95% CI]
Any COVID-19-like symptoms					
	*No*	1	-	-	-
	*Yes*	1.76 [1.19; 2.59]	-	-	-
Timing of COVID-19-likesymptoms					
	*None*	-	1	-	-
	*Before lockdown (<March 17*, *2020)*	-	1.55 [0.97; 2.49]	-	-
	*After lockdown (≥March 17*, *2020)*	-	2.10 [1.18; 3.74]	-	-
Number of COVID-19-like symptoms					
	(Continuous Variable)	-	-	1.22 [1.07; 1.39]	-
Type of COVID-19-likesymptoms					
	*None*	-	-	-	1
	*Mild*	-	-	-	1.93 [1.15; 3.23]
	*Respiratory distress*	-	-	-	2.32 [1.12; 4.81]

**Table 3:** COVID-19-like symptoms and anxiety/depression in the TEMPO cohort during the first COVID-19-related lockdown (March-June 2020, France, multivariate GEE models, adjusted Odds-Ratio (OR), 95% Confidence Interval (CI)).

Variables		Model 1 (n = 656)	Model 2 (n = 655)	Model 3 (n = 623)	Model 4 (n = 623)
		OR [95% CI]	OR [95% CI]	OR [95% CI]	OR [95% CI]
Any COVID-19-like symptoms					
	*No*	1	-	-	-
	*Yes*	1.66 [1.08; 2.55]	-	-	-
Timing of COVID-19-like symptoms					
	*None*	-	1	-	-
	*Before lockdown (<March 17*, *2020)*	-	1.53 [0.90; 2.61]	-	-
	*After lockdown (≥March 17*, *2020)*	-	1.91 [1.03; 3.52]	-	-
Number of COVID-19-like symptoms (Continuous Variable)		-	-	1.19 [1.02; 1.39]	-
Type of COVID-19-like symptoms					
	*None*	-	-	-	1
	*Mild*	-	-	-	1.71 [0.96; 3.05]
	*Respiratory distress*	-	-	-	1.96 [0.93; 4.15]
Sex					
	*Male*	1	1	1	1
	*Female*	1.54 [0.99; 2.38]	1.54 [0.99; 2.38]	1.65 [1.04; 2.61]	1.64 [1.03; 2.59]
Household configuration					
	*Living with a partner + children*	1	1	1	1
	*Living with a partner*	1.36 [0.76; 2.45]	1.35 [0.75; 2.44]	1.56 [0.86; 2.82]	1.57 [0.87; 2.83]
	*Other*	0.79 [0.47; 1.34]	0.79 [0.47; 1.34]	0.74 [0.42; 1.30]	0.75 [0.43; 1.30]
Household income level/month					
	*> 2500€*	1	1	1	1
	*≤ 2500€*	1.89 [1.10; 3.27]	1.89 [1.09; 3.25]	1.92 [1.08; 3.40]	1.90 [1.09; 3.33]

Among covariates, female, low household income, unemployed, diabetes and/or overweight/obesity and anxiety/depression prior to 2020 were also associated with an increase in the odds of anxiety/depression during lockdown.

The only statistical interaction that was statistically significant was between the presence of COVID-19-like symptoms and participants’ sex. After stratifying on sex, we observed a stronger association between COVID-19-like symptoms and anxiety/depression in men than in women (respectively OR = 3.67, 95% CI = 1.39–9.71 and OR = 1.30, 95% CI = 0.80–2.12) (**Table 4**).

**Table 4:** Sex stratified bivariate and multivariate GEE models of anxiety/depression problems and absence/presence of COVID-19-like symptoms.

COVID-19-like symptoms		Males	Females
		OR [95% CI]	OR [95% CI]
**Bivariate model** (Males n = 255 / Female n = 464)			
	No	1	1
	Yes	2.41 [1.07; 5.43]	1.47 [0.93; 2.31]
**Multivariate model** (Males n = 237 / Female n = 419)			
	No	1	1
	Yes	3.67 [1.39; 9.71]	1.30 [0.80; 2.12]

## Discussion

Our study highlights the association between COVID-19-like symptoms and anxiety/depression during the Spring of 2020, which exists even after accounting for individuals’ preexisting mental health difficulties. The higher the number of symptoms, and the more severe the manifestations of COVID-19-like, the higher the odds of anxiety/depression.

This study has some limitations which can impact the interpretation of our results. First, TEMPO participants are a sample of individuals whose parents also participate in a long-term epidemiological study (the GAZEL cohort) [27] and are not representative of the French population. Indeed, due to selective attrition, women are over-represented (65%), as are persons with high socio-economic level, and in good health. Nevertheless, TEMPO participants are a heterogeneous group and sufficiently diverse in terms of geography and socio-economic characteristics to produce generalizable results, but the estimates of associations between COVID-19-like symptoms and anxiety/depression we report may be underestimated. Moreover, levels of mental health difficulties such as depression and anxiety in the TEMPO cohort are comparable to those observed in the general population [28, 29], making TEMPO participants an appropriate sample to study these topics. Second, all data were collected through online questionnaires during lockdown, and participants’ responses were self-reported. Nevertheless, the ASR has been established to evaluate internalizing problems under such conditions [22]. Third, COVID-19-like symptoms were self-reported with no positive PCR test, and misclassification bias is possible. But in the spring of 2020, PCR tests were not widely available in France and there was no possibility to offer a test to study participants [30]. Thus our results suggest that self-reported COVID-19-like symptoms could have a negative impact on mental health.

Our study has several strengths that offset the previously cited limitations. First, the main strength is that anxiety/depression prior to 2020 was collected prospectively before the COVID-19 pandemic, eliminating memory and subjective bias. Second, longitudinal collection of anxiety/depression symptoms in 7 waves during lockdown allowed more consistent results than cross-sectional studies.

The prevalence of anxiety/depression during lockdown of 27% observed in our study is higher than the 18–20% prevalence reported in France in the same period [10]. Differences may be due to differences in scales: the Hospital Anxiety and Depression Scale (HAD) [31] by Santé Publique France and the Adult Self Report (ASR) Achenbach System [22, 23] in our study. In addition, it is important to note that another study using the Mental Health Inventory (MHI-5) indicated that 36% of the French general population presented psychological distress [32]. Our data are also different from those that were reported from other European countries. Indeed, in Germany 45% of individuals were reported to have anxiety symptoms and 14% depression [18], whereas in Italy, 19% of adults reported anxiety and 32% depression [33], these percentages being respectively 20% and 30% in Spain [34] and 22% and 22% in the UK [35]. A meta-analysis reported a prevalence of 32% of anxiety and 34% of depression [5], while literature reviews report a prevalence of anxiety and depression ranging respectively from 6% to 51% and from 15% to 48% [6, 36]. This between-country heterogeneity in prevalence of mental health problems due to COVID-19 infection in the general population may be due to differences in study populations, in the level of confidence towards the government and the setting of the COVID-19 outbreak, but also in differences in terms of scales measuring depression and anxiety used.

The prevalence of mental health difficulties we report is higher than observed outside of the COVID-19 epidemic. Indeed, a recent review suggests that approximately 10% of the French population experiences clinical depression at any one point in time [37] and almost 1% has depression severe enough to require hospitalization [38], which is higher than in most other industrialized countries. In terms of anxiety disorders, approximately 20% of the population is thought to be affected, the most frequent disorders being generalized anxiety disorder (approximately 12%), panic disorder (approximately 6%), social anxiety disorder (approximately 4%), and less than 1% post-traumatic stress disorder [39]. This higher prevalence during the pandemic suggest an impact of COVID-19 and associated preventive measures on mental health [6, 40, 41].

Past studies showed that people who experienced COVID-19 symptoms had an elevated prevalence of mental health problems with an OR ranging from 1.2 to 5.1 for anxiety and from 1.2 to 3.6 for depression [4, 11, 12, 14], which is supported by the findings of the present study even after taking into account prior mental health problems. To study impact of COVID-19, most of studies compared the prevalence of mental health disorders to periods prior to the pandemic [16–18], or with individuals suffering from other forms of pneumonia [42]. But it is essential to take into account confounding factors such as prior mental health symptoms and socio-economic position. When this is the case, in most cases the information was collected retrospectively and may be subject to recall bias. Moreover, as the association between COVID-19 and psychiatric disorder may be directional [43], taking into account history of mental health disorders is crucial. Hence the importance of this longitudinal study.

Nevertheless, our findings suggest that the impact of anxiety/depression prior to 2020 on psychological distress during the COVID-19 lockdown is far greater than the impact of the experience of COVID-19-like symptoms (OR = 5.3 vs 1.7 in model 1 respectively). People with symptoms of anxiety/depression before lockdown are more vulnerable to anxiety/depression during lockdown, which could be explained by the stability and continuity of mental health problems throughout adulthood [44–46]. Moreover, many consequences of COVID-19 pandemic on all aspects of society, including mental health [6, 40, 41], may be increased in vulnerable people.

In our study, women were found to have a higher risk of symptoms of anxiety and depression, which is consistent with prior studies [4, 6, 47, 48]. This may be because in women other factors may contribute to psychological distress, particularly during the COVID-19 epidemic, including increased family and childcare responsibilities, and more job loss and reduced incomes [49–51]. Nevertheless, after stratification on sex, we showed that the relationship between COVID-19-like symptoms and anxiety/depression is stronger among men than women (OR = 3.7 vs 1.3 respectively); to our knowledge this has never previously been shown. Although women in the general population report a higher level of anxiety than men, men are reported to be more anxious in case of chronic pain and men’s catastrophic reactions also have a more negative impact on their mood [52, 53].

The impact of COVID-19-like symptoms on anxiety/depression can be due to several mechanisms. First of all, the fear of being infected, as well as COVID-19 infection or death of close ones could explain the occurrence of stress, anxiety or depression [54]. Second, lockdown leads to disruptions in people’s lives and social isolation [54–56]. Third, economic, social and personal consequences such as job loss, loss of income and financial uncertainty, could also impact mental health problems [40, 57]. A study conducted in France showed that after 10 days of lockdown, 19% of individuals already experienced financial difficulties and 21% were unemployed due to the lockdown [32]. Importantly, these psychological difficulties were observed not only during the course of an epidemic, but also in its aftermath as it was observed in previous epidemics, and could possibly be heightened by feelings of shame and guilt experienced by individuals who have lost close ones, as they could think that they have transmitted the disease, or they may end up being stigmatized because of the epidemic [58].

Another possible mechanism that may explain the onset of mental health problems is the COVID-19 infection itself and its possible biological and immunological impacts. Indeed, SARS-CoV-2 can affect brain tissue by causing a cytokine storm, which is believed to have an impact on neurological and psychiatric symptoms, and trigger an immune response that could have an impact on mental health [59, 60]. During this cytokine storm, pro-inflammatory factors are intensively released and enter the central nervous system (CNS), initiating a neuro-inflammatory process [61]. Moreover, an excessive and dysregulated immune response seen in patients affected by COVID-19 contribute to high levels of various pro-inflammatory cytokines, these cytokines are elevated in patients with depression and could be a hypothetical mechanism distinct from social isolation and psycho-socioeconomic stressors [60]. Additionally, the presence of the SARS-Cov-2 virus in the brain can also manifest in psychiatric symptoms [59]. Some biological alterations due to coronavirus infection were found, especially activation of microglia and cytokine signaling, that are common alterations in psychotic disorders without establishing a causal relationship [59].

The association between the number of COVID-19-like symptoms and the odds of anxiety/depression could be explained by fear related to symptoms severity, and also the increased biological alterations described above.

## Conclusions

Our study shows elevated levels of symptoms of anxiety/depression among persons who experienced COVID-19-like symptoms, even after accounting for mental health difficulties prior to 2020. This suggests that COVID-19 infection may have both a direct, virus itself, and indirect, consequences of the epidemic, impact on psychological difficulties, which should be studied in greater detail.

## Supporting information

S1 Table (DOCX)

S2 Table (DOCX)

S3 Table (DOCX)
