# Supplementary material for: Impact of COVID-19-like symptoms on occurrence of anxiety/depression during lockdown among the French general population
Source: PLoS One. 2021 Jul 26;16(7):e0255158. doi: 10.1371/journal.pone.0255158 (PMC8312967; doi:10.1371/journal.pone.0255158)
Supplement: S1 Table — (DOCX) [file pone.0255158.s001.docx]

**Supplementary Table 1. TEMPO Covid-19 collection**

|  |  | **Wave** | | | | | | |
| --- | --- | --- | --- | --- | --- | --- | --- | --- |
|  |  | **1** | **2** | **3** | **4** | **5** | **6** | **7** |
| Period of data collection | | 24/03 - 31/03 | 31/03 - 07/04 | 07/04 - 14/04 | 14/04 - 21/04 | 21/04 - 05/05 | 05/05 - 19/05 | 19/05 - 02/06 |
| Number of participants | | 424 | 544 | 493 | 466 | 428 | 420 | 386 |
|  |  |  |  |  |  |  |  |  |
