# Supplementary material for: Impact of COVID-19-like symptoms on occurrence of anxiety/depression during lockdown among the French general population
Source: PLoS One. 2021 Jul 26;16(7):e0255158. doi: 10.1371/journal.pone.0255158 (PMC8312967; doi:10.1371/journal.pone.0255158)
Supplement: S2 Table — (DOCX) [file pone.0255158.s002.docx]

**Supplementary Table 2. Adult Self Report (ASR) anxiety/depression scale: items used**

| Wave 1 (8 items) | Wave 2 (13 items) |
| --- | --- |
| I cry a lot | I cry a lot |
| I worry about my future | I worry about my future |
| I feel worthless or inferior | I feel worthless or inferior |
| I am nervous or tense | I am nervous or tense |
| I feel too guilty | I feel too guilty |
| I think about killing myself | I think about killing myself |
| I am unhappy, sad, or depressed | I am unhappy, sad, or depressed |
| I feel that I can't succeed | I feel that I can't succeed |
|  | I am afraid I might think or do something bad |
|  | I feel that no one loves me |
|  | I feel that others are out to get me |
|  | I am self-conscious or easily embarrassed |
|  | I worry about my social relations with the opposite sex |
