# Supplementary material for: Impact of COVID-19-like symptoms on occurrence of anxiety/depression during lockdown among the French general population
Source: PLoS One. 2021 Jul 26;16(7):e0255158. doi: 10.1371/journal.pone.0255158 (PMC8312967; doi:10.1371/journal.pone.0255158)
Supplement: S3 Table — (DOCX) [file pone.0255158.s003.docx]

**Supplementary Table 3. Association between COVID-19-like symptoms and anxiety/depression taking into account prior anxiety/depression symptoms with different definitions (multivariate GEE models, adjusted Odds-Ratio (OR), 95% Confidence Interval (CI))**

|  | **Prior anxiety/depression**  **(most recent information)**  OR [95% CI] | **Prior anxiety/depression**  **(oldest information)**  OR [95% CI] |
| --- | --- | --- |
| Any COVID-19-like symptoms  *No*  *Yes* | 1  1.66 [1.08 ; 2.55] | 1  1.55 [1.00 ; 2.39] |
| Timing of COVID-19-like symptoms  *None*  *Before lockdown (<March 17, 2020)*  *After lockdown (≥March 17, 2020)* | 1  1.53 [0.90 ; 2.61]  1.91 [1.03 ; 3.52] | 1  1.30 [0.73 ; 2.31]  1.94 [1.10 ; 3.43] |
| Number of COVID-19-like symptoms  *(Continuous Variable)* | 1.19 [1.02 ; 1.39] | 1.20 [1.02 ; 1.40] |
| Type of COVID-19-like symptoms  *None*  *Mild*  *Respiratory distress* | 1  1.71 [0.96 ; 3.05]  1.96 [0.93 ; 4.15] | 1  1.59 [0.91 ; 2.78]  1.95 [0.89 ; 4.29] |
